# Supplementary material for: MiREDiBase, a manually curated database of validated and putative editing events in microRNAs
Source: Sci Data. 2021 Aug 4;8:199. doi: 10.1038/s41597-021-00979-8 (PMC8338996; doi:10.1038/s41597-021-00979-8)
Supplement: Supplementary file 1 — Supplementary Materials [file 41597_2021_979_MOESM1_ESM.pdf]

MiREDiBase, a manually curated database of editing events in microRNAs

Gioacchino P. Marceca[1†], Rosario Distefano[2†], Luisa Tomasello[2], Alessandro Lagana'[3], Francesco Russo[4], Federica Calore[2], Giulia Romano[5], Marina Bagnoli[6], Pierluigi Gasparini[2,7,8], Alfredo Ferro[1], Mario Acunzo[5], Qin Ma[9], Carlo M. Croce[2‡] and Giovanni Nigita[2‡*]

Affiliations
1. Department of Clinical and Experimental Medicine, University of Catania, Catania, Italy.
2. Department of Cancer Biology and Genetics and Comprehensive Cancer Center, The Ohio State University, Columbus, OH, USA.
3. Department of Genetics and Genomic Sciences, Icahn School of Medicine at Mount Sinai, New York, NY, USA.
4. Section for Clinical Mass Spectrometry, Danish Center for Neonatal Screening, Department of Congenital Disorders, Statens Serum Institut, Copenhagen, Denmark
5. Division of Pulmonary Diseases and Critical Care Medicine, Virginia Commonwealth University, Richmond, VA, USA.
6. Fondazione IRCCS Istituto Nazionale dei Tumori (INT), Milan, Italy.
7. School of Biomedical Sciences and Pharmacy, College of Health, Medicine and Wellbeing, University of Newcastle, Callaghan, NSW, Australia.
8. Hunter Medical Research Institute, New Lambton Heights, NSW, Australia.
9. Department of Biomedical Informatics, College of Medicine, The Ohio State University, Columbus, OH, USA.

*Corresponding Authors:

Carlo M. Croce (carlo.croce@osumc.edu)

Giovanni Nigita (giovanni.nigita@osumc.edu)

† These authors have contributed equally to this work
‡ These authors have contributed equally to this work

TABLE CONTENTS

Supplementary Figures…………………………………………………………………………...… 2-6
Supplementary Figure 1. Validated and putative miRNA editing events in primates. Total number of validated and putative A-to-I and C-to-U editing sites per primate species. ……………………………………………. 2
Supplementary Figure 2. Descriptive statistics about macaque data in the current version of MiREDiBase. ………… 3
Supplementary Figure 3. Validated and putative miRNA editing event distribution in miRNA sequences. …………... 4
Supplementary Figure 4. Nucleotide distribution analysis of nearest neighbors of human and macaque miRNA edited adenosines. ……………………………………………………………………………………………... 5
Supplementary Figure 5. Overview of the MiREDiBase multi-containerized microservice architecture. ……………... 6

Supplementary Tables …………………………………………………………………………….. 7-18
Supplementary Table 1. List of articles included in the current version of MiREDiBase. …………………………….. 7
Supplementary Table 2. List of healthy human tissues included in the current version of MiREDiBase. ….…………… 10
Supplementary Table 3. List of pathological conditions in humans included in the current version of MiREDiBase. .. 12
Supplementary Table 4. List of human cell lines included in the current version of MiREDiBase. …………………... 15
Supplementary Table 5. List of healthy tissues in Primates included in the current version of MiREDiBase. ….. 18

Supplementary Data Set Legend ………………………………………………………………………19

SUPPLEMENTARY FIGURES

Supplementary Figure 1

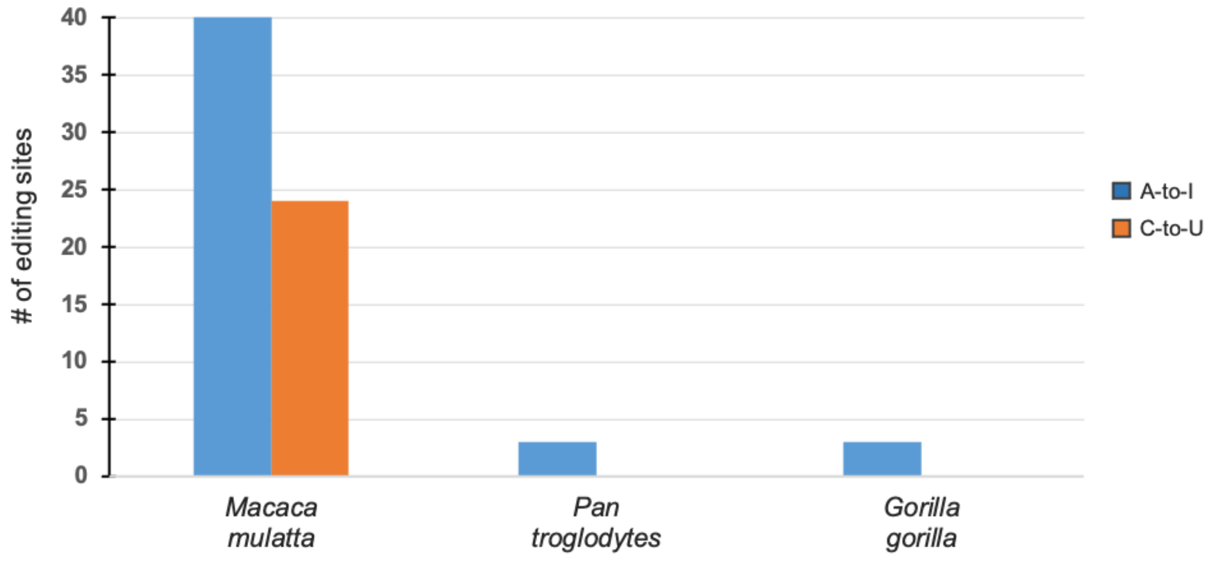

Supplementary Figure 1. **Validated and putative miRNA editing events in primates**. Total number of validated and putative A-to-I and C-to-U editing sites per primate species.

Supplementary Figure 2

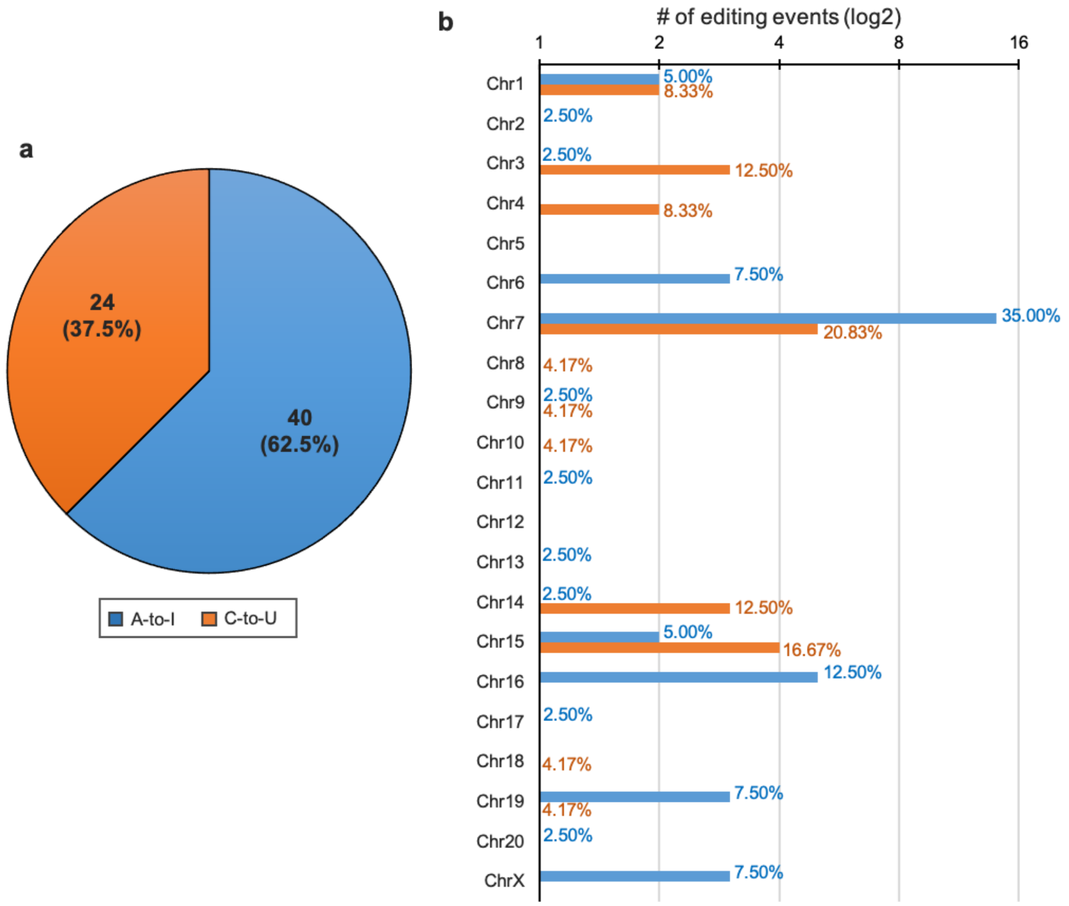

Supplementary Figure 2. **Descriptive statistics about macaque data in the current version of MiREDiBase**. (a) Pie chart showing the total number of unique validated and putative A-to-I and C-to-U editing events reported from macaque tissues. (b) Distribution of validated and putative A-to-I and C-to-U miRNA editing events per chromosome in the macaque genome.

Supplementary Figure 3

a

	Total no. of macaque miRNA editing events	# of editing events in pri-miRs	# of editing events in pre-miRs	# of editing events in mature miRs
A-to-I	40	0	5	35
C-to-U	24	0	2	22

b

	1	2	3	4	5	6	7	8	9	10	11	12	13	14	15	16	17	18	19	20	21	22	23
# of A-to-I EEs	0	2	2	3	3	9	1	1	2	4	0	0	1	0	0	2	3	0	0	2	0	0	0
# of C-to-U EEs	0	0	0	0	0	0	0	1	0	6	5	3	2	0	2	1	2	0	0	0	0	0	0
Position in miRs	1	2	3	4	5	6	7	8	9	10	11	12	13	14	15	16	17	18	19	20	21	22	23
		Seed region																					

min max

Supplementary Figure 3. Validated and putative miRNA editing event distribution in miRNA sequences. (a) Distribution of validated and putative A-to-I and C-to-U editing events across the three different regions of primary miRNA transcripts. (b) Distribution of editing sites falling into mature miRNAs across nucleotide positions.

Supplementary Figure 4

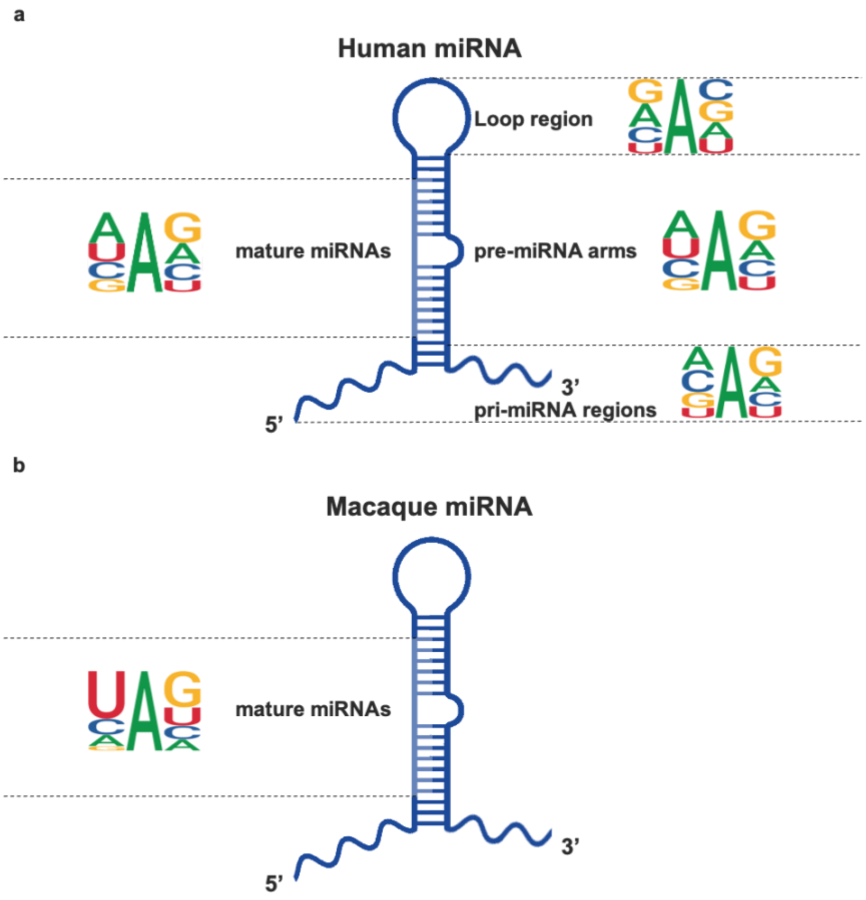

Supplementary Figure 4. Nucleotide distribution analysis of nearest neighbors of human and macaque miRNA edited adenosines. Nucleotide distribution around the editing sites across the different regions of (a) human and (b) macaque miRNA transcripts. In each sequence logo, the As, at the second position, represent the adenosines that undergo RNA editing.

Supplementary Figure 5

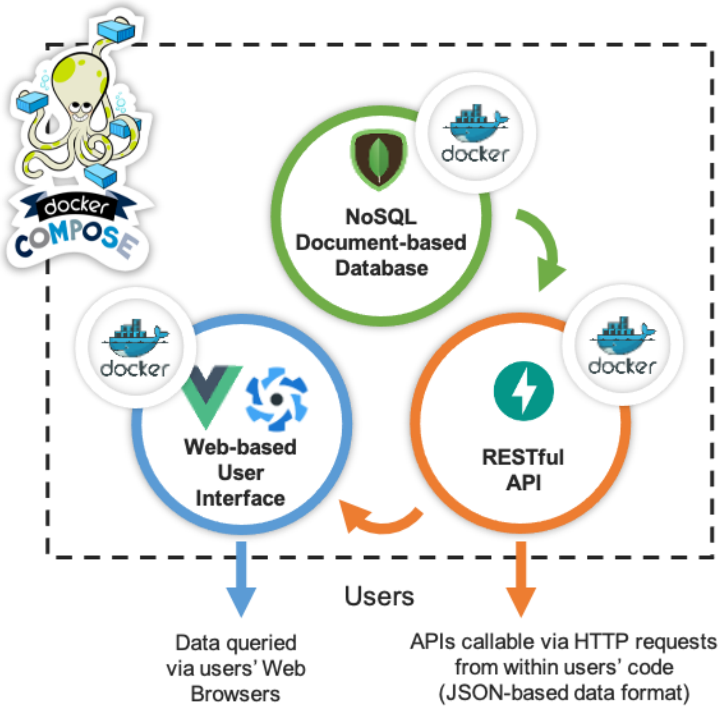

Supplementary Figure 5. Overview of the MiREDiBase multi-containerized microservice architecture. The MiREDiBase platform provides users different ways to access its data: through a web browser (Web-based User Interface) and RESTful API (HTTP calls).

SUPPLEMENTARY TABLES

Supplementary Table 1

First author	Year of publication	Title	Journal	PMID	Approach*
Kim et al.	2004	Widespread RNA editing of embedded alu elements in the human transcriptome	Genome Research	15342557	2
Luciano et al.	2004	RNA editing of a miRNA precursor	RNA	15272117	1
Blow et al.	2006	RNA editing of human microRNAs	Genome Biology	16594986	1
Kawahara et al. (a)	2007	Redirection of silencing targets by adenosine-to-inosine editing of miRNAs	Science	17322061	1
Kawahara et al. (b)	2007	RNA editing of the microRNA-151 precursor blocks cleavage by the Dicer-TRBP complex	EMBO Reports	17599088	1
Landgraf et al.	2007	A mammalian microRNA expression atlas based on small RNA library sequencing	Cell	17604727	2
Kawahara et al.	2008	Frequency and fate of microRNA editing in human brain	Nucleic Acid Reseach	18684997	1
Li et al.	2009	Genome-wide identification of human RNA editing sites by parallel DNA capturing and sequencing	Science	19478186	2
de Hoon et al.	2010	Cross-mapping and the identification of editing sites in mature microRNAs in high-throughput sequencing libraries	Genome Research	20051556	2
Martì et al.	2010	A myriad of miRNA variants in control and Huntington's disease brain regions detected by massively parallel sequencing	Nucleic Acid Reseach	20591823	2
Skalsky and Cullen	2011	Reduced expression of brain-enriched microRNAs in glioblastomas permits targeted regulation of a cell death gene	PLoS One	21912681	2
Bahn et al.	2012	Accurate identification of A-to-I RNA editing in human by transcriptome sequencing	Genome Research	21960545	2
Peng et al.	2012	Comprehensive analysis of RNA-Seq data reveals extensive RNA editing in a human transcriptome	Nature Biotechnology	22327324	2
Ramaswami et al.	2012	Accurate identification of human Alu and non-Alu RNA editing sites	Nature Methods	22484847	2
Alon et al.	2012	Systematic identification of edited microRNAs in the human brain	Genome Research	22499667	2
Choudhury et al.	2012	Attenuated adenosine-to-inosine editing of microRNA-376a* promotes invasiveness of glioblastoma cells	Journal of Clinical Investigation	23093778	1, 2
Chen et al.	2013	Characterization and comparison of human nuclear and cytosolic editomes	Proceedings of the National Academy of Sciences	23818636	2
Ramaswami et al.	2013	Identifying RNA editing sites using RNA sequencing data alone	Nature Methods	23291724	2
Liu et al.	2013	ADAR2-mediated editing of miR-214 and miR-122 precursor and antisense RNA transcripts in liver cancers	PLoS One	24386085	1
Bazak et al.	2014	A-to-I RNA editing occurs at over a hundred million genomic sites, located in a majority of human genes	Genome Research	24347612	2
Warnefors et al.	2014	Conserved microRNA editing in mammalian evolution, development and disease	Genome Biology	24964909	2
Porath et al.	2014	A genome-wide map of hyper-edited RNA reveals numerous new sites	Nature Communications	25158696	2

Zheng et al.	2014	Revealing editing and SNPs of microRNAs in colon tissues by analyzing high-throughput sequencing profiles of small RNAs	BMC Genomics	25521855	2
Gong et al.	2014	Comprehensive analysis of human small RNA sequencing data provides insights into expression profiles and miRNA editing	RNA Biology	25692236	2
Tomaselli et al.	2015	Modulation of microRNA editing, expression and processing by ADAR2 deaminase in glioblastoma	Genome Biology	25582055	2
Shoshan et al.	2015	Reduced adenosine-to-inosine miR-455-5p editing promotes melanoma growth and metastasis	Nature Cell Biology	25686251	1, 2
Negi et al.	2015	Altered expression and editing of miRNA-100 regulates iTreg differentiation	Nucleic Acid Reseach	26209130	1, 2
Picardi et al.	2015	Profiling RNA editing in human tissues: towards the inosinome Atlas	Scientific Reports	26449202	2
Saiselet et al.	2015	New global analysis of the microRNA transcriptome of primary tumors and lymph node metastases of papillary thyroid cancer	BMC Genomics	26487287	1, 2
Yuan et al.	2016	Variability of miRNA expression during the differentiation of human embryonic stem cells into retinal pigment epithelial cells	Gene	26028588	2
Anadón et al.	2016	Gene amplification-associated overexpression of the RNA editing enzyme ADAR1 enhances human lung tumorigenesis	Oncogene	26640150	1
Zheng et al.	2016	Accurate detection for a wide range of mutation and editing sites of microRNAs from small RNA high-throughput sequencing profiles	Nucleic Acid Reseach	27229138	2
Zipeto et al.	2016	ADAR1 Activation Drives Leukemia Stem Cell Self-Renewal by Impairing Let-7 Biogenesis	Cell Stem Cell	27292188	1, 2
Nigita et al.	2016	microRNA editing in seed region aligns with cellular changes in hypoxic conditions	Nucleic Acid Reseach	27298257	2
Picardi et al.	2017	REDIportal: a comprehensive database of A-to-I RNA editing events in humans	Nucleic Acid Reseach	27587585	2
Wang et al.	2017	Systematic characterization of A-to-I RNA editing hotspots in microRNAs across human cancers	Genome Research	28411194	1, 2
Paul et al.	2017	A-to-I editing in human miRNAs is enriched in seed sequence, influenced by sequence contexts and significantly hypoedited in glioblastoma multiforme	Scientific Reports	28550310	2
Gallego et al.	2017	RNA editing independently occurs at three mir-376a-1 sites and may compromise the stability of the microRNA hairpin	Gene	28710037	1, 2
D'Erchia et al.	2017	Massive transcriptome sequencing of human spinal cord tissues provides new insights into motor neuron degeneration in ALS	Scientific Reports	28855684	2
Daniel et al.	2017	Editing inducer elements increases A-to-I editing efficiency in the mammalian transcriptome	Genome Biology	29061182	1
Cesarini et al.	2017	ADAR2/miR-589-3p axis controls glioblastoma cell migration/invasion	Nucleic Acid Reseach	29267965	1, 2
Pinto et al.	2018	Human cancer tissues exhibit reduced A-to-I editing of miRNAs coupled with elevated editing of their targets	Nucleic Acid Reseach	29165639	2
Li et al.	2018	The landscape of miRNA editing in animals and its impact on miRNA biogenesis and targeting	Genome Research	29233923	2
van der Kwast et al.	2018	Adenosine-to-Inosine Editing of MicroRNA-487b Alters Target Gene Selection After Ischemia and Promotes Neovascularization	Circulation Research	29284691	1
Velazquez-Torres et al.	2018	A-to-I miR-378a-3p editing can prevent melanoma progression via regulation of PARVA expression	Nature Communications	29386624	1

Ishiguro et al.	2018	Base-pairing probability in the microRNA stem region affects the binding and editing specificity of human A-to-I editing enzymes ADAR1-p110 and ADAR2	RNA Biology	29950133	2
Nigita et al.	2018	Tissue and exosomal miRNA editing in Non-Small Cell Lung Cancer	Scientific Reports	29976955	2
Maemura et al.	2018	Altered editing level of microRNAs is a potential biomarker in lung adenocarcinoma	Cancer Science	30022565	1, 2
Jiang et al.	2019	Hyper-Editing of Cell-Cycle Regulatory and Tumor Suppressor RNA Promotes Malignant Progenitor Propagation	Cancer Cell	30612940	1
Xu et al.	2019	A-to-I-edited miRNA-379-5p inhibits cancer cell proliferation through CD97-induced apoptosis	Journal of Clinical Investigation	31682236	2
Wang et al.	2019	Identifying microRNAs and Their Editing Sites in Macaca mulatta	Cells	31284505	2

Supplementary Table 1. **List of articles included in the current version of MiREDiBase**. The list includes first author, year of publication, article's title, journal, Pubmed ID, and approach used to identify editing sites. * 1 = targeted approach; 2 = wide-transcriptome approach

Supplementary Table 2

Anatomical area/Tissue	Anatomical subdivision/ Developmental stage
Adipose tissue	adipose tissue (subcutaneous)<br>adipose tissue (visceral)
Adrenal gland	--
Artery	--
Bladder	--
Blood	blood (cord blood)<br>blood (hematopoietic stem cells)<br>blood (lymphoblastoid cells)<br>blood (mononuclear cells)<br>umbilical cord (mesenchymal stem cells)
Bone Marrow	--
Brain	brain (amygdala)<br>brain (anterior cingulate cortex)<br>brain (caudate nucleus)<br>brain (cerebellum)<br>brain (corpus callosum)<br>brain (corpus striatum)<br>brain (cortex)<br>brain (fetal)<br>brain (frontal cortex)<br>brain (frontal gyrus)<br>brain (frontal lobe)<br>brain (hippocampus)<br>brain (hypothalamus)<br>brain (medulla oblongata)<br>brain (midbrain)<br>brain (nucleus accumbens)<br>brain (pituitary gland)<br>brain (prefrontal cortex)<br>brain (putamen)<br>brain (substantia nigra)
Breast	breast (mammary gland)<br>breast (milk)
Epididymis	--
Esophagus	mucosa<br>smooth muscle
Eye	eye (retinal pigment epithelium) (fetal)
Fibroblasts	--
Head-Neck	--
Heart	--
Intestine	intestine (colon)<br>intestine (rectum)

	intestine (small)<br>intestine (stomach)
Kidney	--
Leucocytes	leucocytes (dendritic cells)<br>leucocytes (granulocytes)<br>leucocytes (macrophages M0)<br>leucocytes (monocytes)
Liver	liver (bile duct)<br>liver (fetal)
Lung	--
Lymph node	lymphocytes (B-cells)<br>lymphocytes (T-cells)
Ovary	--
Pancreas	--
Placenta	--
Prostate	--
Salivary gland	--
Skeletal muscle	--
Skin	skin (mucosa)
Spinal cord	--
Spleen	spleen (fetal)
Testis	testis (seminal liquid)
Thymus	--
Thyroid	--
Uterus	uterus (cervical)<br>uterus (fallopian tube)
Vagina	--

Supplementary Table 2. List of healthy human tissues included in the current version of MiREDiBase. Human organs and tissues in which editing events have been observed are listed in the "Anatomical area/Tissue" column. Eventual organs' anatomical subdivisions (e.g., brain anatomical subdivisions) or prenatal developmental stages are indicated in "Anatomical subdivision/Developmental stage".

Supplementary Table 3

Disease typology	Disease	Specification
**Oncological disease**	ACC = adrenocortical carcinoma	--
Oncological disease	ALL = acute lymphocytic leukemia	ALL (B-cells)
		ALL (T-cells)
Oncological disease	AML = acute myeloid leukemia	--
Oncological disease	AS = astrocytoma	--
Oncological disease	ASBL = astroblastoma	--
Oncological disease	BL = Burkitt lymphoma	--
Oncological disease	BLCA = bladder carcinoma	--
Oncological disease	BRCA = breast cancer	--
Oncological disease	CEAD = cervical adenocarcinoma	--
Oncological disease	CESC = cervical squamous cell carcinoma and endocervical adenocarcinoma	--
Oncological disease	CHOL = cholangiocarcinoma	--
Oncological disease	CLL = chronic lymphoid leukemia	--
Oncological disease	CM = cutaneous melanoma	--
Oncological disease	CML = chronic myeloid leukemia	--
Oncological disease	COAD = colon adenocarcinoma	--
Oncological disease	CRC = colorectal carcinoma	--
Oncological disease	DLBCL = diffuse large B cell lymphoma	--
Oncological disease	EBT = embryonal brain tumor	EBT (not defined)
Oncological disease	ESCA = Esophageal carcinoma	--
Oncological disease	FL = follicular lymphoma	--
Oncological disease	GBM = glioblastoma	--
Oncological disease	GM = glioma	GM (not defined)
Oncological disease	HCC = hepatocellular carcinoma	--
Oncological disease	HL = Hodgkin lymphoma	--
Oncological disease	HNSC = head and neck squamous cell carcinoma	--
Oncological disease	KICH = kidney chromophobe	--
Oncological disease	KIRC = kidney renal clear cell carcinoma	--
Oncological disease	KIRP = kidney renal papillary cell carcinoma	--
Oncological disease	LGG =low grade glioma	--
Oncological disease	LNM = lymph node metastasis	--
Oncological disease	LUAD = lung adenocarcinoma	--
Oncological disease	LUSC = lung squamous cell carcinoma	--
Oncological disease	MB = medulloblastoma	--
Oncological disease	MCC = Merkel cell carcinoma	--
Oncological disease	MESO = mesothelioma	--
Oncological disease	MM = multiple myeloma	--

Oncological disease	NB = neuroblastoma	--
Oncological disease	NHL = other types of non-Hodgkin lymphoma	NHL (B-cells)
		NHL (T-cells)
Oncological disease	NSCLC = non-small cell lung cancer	--
Oncological disease	ODGM = oligodendroglioma	--
Oncological disease	OS = osteosarcoma	--
Oncological disease	OVAD = ovarian adenocarcinoma	--
Oncological disease	OVCA = ovarian cancer	--
Oncological disease	PAAD = pancreatic adenocarcinoma	--
Oncological disease	PCPG = pheochromocytoma and paraganglioma	--
Oncological disease	PRAD = prostate adenocarcinoma	--
Oncological disease	PTC = papillary thyroid cancer	--
Oncological disease	RCAD = renal cellular adenocarcinoma	--
Oncological disease	RCC = renal cellular carcinoma	--
Oncological disease	READ = rectum adenocarcinoma	--
Oncological disease	SARC = sarcoma	--
Oncological disease	SEMIN = seminoma	--
Oncological disease	STAD = stomach adenocarcinoma	--
Oncological disease	TC = testicular cancer	--
Oncological disease	TERATO = teratocarcinoma	--
Oncological disease	THCA = thyroid carcinoma	--
Oncological disease	THYM = thymoma	--
Oncological disease	UCEC = uterine corpus endometrial carcinoma	--
Oncological disease	UCS = uterine carcinosarcoma	--
Oncological disease	UVM = uveal melanoma	--
**Neurological disorders**	Alzheimer disease	Alzheimer disease (prefrontal cortex)
Neurological disorders	Amyotrophic lateral sclerosis (ALS)	Amyotrophic lateral sclerosis (ALS) (spinal cord)
Neurological disorders	Huntington disease	Huntington disease (brain (corpus striatum))
		Huntington disease (brain (frontal cortex))
		Huntington disease (prefrontal cortex)
Neurological disorders	Parkinson disease	Parkinson disease (prefrontal cortex)

**Genetic disorders**	Down syndrome	Down syndrome (cord blood mononuclear cells)
**Inflammatory / Autoimmune condition**	Hepatitis	hepatitis B (liver)
		hepatitis C (liver)
Inflammatory / Autoimmune condition	Skin psoriasis	skin psoriasis (skin)
**Cardiovascular disease**	Ischemia	ischemia (heart)

Supplementary Table 3. **List of pathological conditions in humans included in the current version of MiREDiBase**.

Diseases and disorders listed in “Disease” are classified into pathological categories (Disease typology).

Supplementary Table 4

Condition / Malignancy	Cell line	Specification
**Artery**	HUAF (primary)	--
Artery	HUASMC (primary)	--
Artery	HUVEC (primary)	--
**Brain**	neurons (iPSC-derived)	--
Brain	normal astrocytes	--
**Embryonic Stem Cells**	BG01	--
Embryonic Stem Cells	cord blood (HSPC)	--
Embryonic Stem Cells	H9	--
Embryonic Stem Cells	hESCs	--
Embryonic Stem Cells	hiPS2	--
Embryonic Stem Cells	HSF1	--
**Fibroblasts**	WI38	--
**Kidney (embryonic)**	HEK293	HEK293<br>HEK293 (transfected)*
**Lymphocytes (B-cells)**	lymphoblastoid cell line (B-cells)	--
**Lymphocytes (T-cells)**	A301	--
**Skin**	normal melanocytes	--
**ACC**	H295R	--
ACC	SW13	--
**AML**	HL60	--
AML	Kas1	--
AML	THP1	--
**AS**	SW1088	--
AS	SW1783	--
**BL**	AKBM	--
BL	BL41	--
BL	CA46	--
BL	DG75	--
BL	HK1	--
BL	Jijoye	--
BL	Raji	--
**BRCA**	BT474	--
BRCA	HCC38	--
BRCA	Hs578T	Hs578T<br>Hs578T (transfected)*
BRCA	MCF10A	--

BRCA	MCF7	--
BRCA	MDAMB231	--
BRCA	SKBR3	--
BRCA	T47D	--
**CEAD**	HeLa	HeLa<br>HeLa (transfected)*
**CLL**	EHEB	--
CLL	Mec2	--
**CM**	A375	--
CM	C8161	C8161<br>C8161 (transfected)*
CM	SB2	--
**CML**	cord blood (HSPC) (CML)	cord blood (HSPC) (CML)<br>cord blood (HSPC) (CML) (transfected)*
CML	K562	--
**COAD**	DKS8	--
COAD	DLD1	--
**CRC**	HCT116	--
**DLBCL**	OCI-LY1	--
DLBCL	SUDHL6	--
**GBM**	A172	A172<br>A172 (transfected)*
GBM	M059K	M059K (transfected)*
GBM	SNB19	--
GBM	U118	U118<br>U118 (transfected)*
GBM	U251	--
GBM	U87	U87<br>U87 (transfected)*
**HCC**	HepG2	--
HCC	Huh7	--
HCC	PLC	--
**HL**	KMH2	--
HL	L1236	--
HL	L428	--
HL	L591	--
**LUAD**	A549	--
LUAD	H1568	--
LUAD	H1993	--
LUAD	H23	--
**LUSC**	H520	--

**MB**	DAOY	--
**MM**	U266	--
**NB**	BE2	--
NB	SHSY5Y	--
**NHL (B-cells)**	BCBL1	--
NHL (B-cells)	Granta519	--
NHL (B-cells)	Jeko1	--
NHL (B-cells)	JVM2	--
NHL (B-cells)	LCL-BACD1	--
**NHL (T-cells)**	Sup-T1	--
**OS**	A673	--
OS	U2OS	--
**OVAD**	HeyA8	HeyA8<br>HeyA8 (transfected)*
**OVCA**	OVCAR8	--
**RCAD**	786O	786O<br>786O (transfected)*
**RCC**	DH1	--
**SEMIN**	NCCIT	--
**TERATO**	NT2	--
TERATO	PA1	--

Supplementary Table 4. List of human cell lines included in the current version of MiREDiBase. The table shows what condition each cell line represents (Condition /Malignancy). *The wording "transfected" is referred only to cell lines transfected with a vector overexpressing wild type ADAR1/ADAR2.

Supplementary Table 5

Macaque

Anatomical area/Tissue	Anatomical subdivision/ Developmental stage
Blood	blood (mononuclear cells)
Bone marrow	--
Brain	brain - neurons (progenitor cells)
	brain (cerebellum)
	brain (cortex)
	brain (frontal cortex)
	brain (occipital lobe)
	brain (pituitary gland)
	brain (prefrontal cortex)
Embryo	embryonic stem cells
Heart	--
Intestine	intestine (large)
	intestine (rectum)
	intestine (small)
Kidney	--
Liver	--
Lung	--
Lymph node	Lymphoblastoid cell line
Skeletal muscle	--
Spinal cord	--
Spleen	--
Testis	--
Uterus	uterus (endometrium)

Chimpanzee

Anatomical area/Tissue	Anatomical subdivision/ Developmental stage
Brain	brain (cortex)

Gorilla

Anatomical area/Tissue	Anatomical subdivision/ Developmental stage
Brain	brain (cortex)

Supplementary Table 5. **List of healthy tissues in Primates included in the current version of MiREDiBase**. Primate organs and tissues in which editing events have been observed are listed in the "Anatomical area/Tissue" column. Eventual organs' anatomical subdivisions (e.g., brain anatomical subdivisions) or prenatal developmental stages are indicated in "Anatomical subdivision/Developmental stage".

SUPPLEMENTARY DATA SET LEGENDS

Supplementary Data Set 1. **List of unique validated and putative A-to-I and C-to-U miRNA editing events in humans and primates included in the current version of MiREDiBase**. Editing events concerning mature miRNAs are highlighted in green. For each editing event, the number of reports in the current literature is reported, with relative Pubmed IDs.
